# Immunization status and its determinants among children aged 12–23 months at community health centers in Mogadishu, Somalia: a cross-sectional study

**DOI:** 10.3389/fped.2025.1504255

**Published:** 2025-05-07

**Authors:** Shafie Abdulkadir Hassan, Abdifatah Abdullahi Abukar, Abdulahi Salad Gutale, Asma Isak Hassan, Abdulahi Jabril Haji, Abdulkadir Mohamed Nur, Mohamed Ibrahim Adam, Ahmed Nor Osman Hassan, Mowlid Abdikarin Mohamed, Abdifetah Ibrahim Omar, Nur Rashid Ahmed

**Affiliations:** ^1^ Faculty of Medicine and Health Science, Jamhuriya University of Science and Technology, Mogadishu, Somalia; ^2^ Jamhuriya Research Center, Jamhuriya University of Science and Technology, Mogadishu, Somalia

**Keywords:** vaccination status, determinants, children, community health centers, Somalia

## Abstract

**Background:**

Routine vaccination is essential in reducing child mortality. This study assessed the determinants of vaccination status and its determinants for children aged 12–23 months in community health centers in Mogadishu, Somalia.

**Methods:**

The study involved 417 mothers, systematically selected from those attending community health centers in Mogadishu for antenatal care between March 25 and June 15, 2024. Data was collected through a structured questionnaire, and SPSS was used for the analysis. Logistic regression analyses of both bivariate and multivariate were conducted to examine the association between dependent and independent variables. The findings were reported as adjusted odds ratios (AOR) with 95% confidence intervals, and a *p*-value of less than 0.05 determined statistical significance.

**Results:**

Vaccination rates showed that 53% of children were fully vaccinated, while 47% had received partial vaccinations. Findings revealed that higher household income was associated with increased odds of partial vaccination status, with families earning $300–$400 monthly being most likely to be partially vaccinated (AOR: 3.247, 95% CI: 1.784–5.910, *p* = 0.002). Additionally, children whose mothers had no antenatal care (ANC) visits were significantly less likely to be fully vaccinated (AOR = 20.075, 95% CI: 7.385–54.572, *p* = 0.001) compared to those whose mothers attended four or more ANC visits.

**Conclusion:**

Our findings revealed that 53% of children were fully vaccinated, with higher household income and regular antenatal care visits associated with increased vaccination rates. Future research should focus on interventions to improve maternal health services and increase access to antenatal care (ANC) to raise vaccination rates in Somalia. Further investigation should explore barriers to vaccination in low-income areas and innovative community engagement strategies.

## Introduction

Immunization coverage refers to the proportion of children who have received essential vaccinations to protect them from illnesses (1). Africa carries the highest burden of under-five mortality globally, with Sub-Saharan Africa experiencing a rate of 74 deaths per 1,000 live births, compared to the global average of 37 per 1,000 live births (2). A significant proportion of these deaths are attributed to preventable infectious diseases, such as pneumonia, diarrhea, malaria, and measles (3), despite the availability of effective vaccines; measles alone, a highly preventable disease, remains a substantial cause of childhood mortality even though the vaccine has a 95%–97% efficacy when two doses are administered (4). Even with advancements in vaccine accessibility through the WHO's Expanded Programme on Vaccination (EPI), many African countries continue to experience low vaccination rates (5). The situation is particularly severe in Sub-Saharan Africa, where many countries face significant challenges in achieving global vaccination targets (6, 7). Although progress has been made over the past few decades, routine vaccination coverage remains below the desired level in many nations (8). In Somalia, the infant mortality rate is exceedingly high, with approximately 81 deaths per 1,000 live births, primarily attributable to preventable illnesses, including measles, which contributes to approximately 30% of childhood mortality (2, 9).

In Somalia, decades of conflict, political instability, and a weakened healthcare system have created a particularly challenging vaccination landscape, resulting in coverage that remains significantly lower than in many other parts of Africa (10–12). Recent studies from 2020 to 2023 conducted in various locations across Somalia indicate that only about 20%–55% of children receive all necessary vaccines, with measles and polio continuing to be endemic in certain regions (1, 13, 14). Previous indicated that higher household income is linked to improved access to healthcare, including vaccinations (14, 15). Conversely, several studies have associated maternal literacy with better vaccination coverage (1, 13, 14, 16–19). Finally, Studies indicate that ANC visits significantly improve childhood vaccination rates through maternal education, care access, and vaccination schedule adherence (13, 20).

Despite ongoing efforts by the Ministry of Health, along with support from international donors such as WHO, UNICEF, and GAVI, to increase vaccination rates in Somalia to increase vaccination rates, many children remain vulnerable to vaccine-preventable diseases in Somalia, leading to increased morbidity and mortality rates (10, 21). While some research has examined barriers to vaccination, there is a lack of detailed studies focused on Somalia. Our study is essential as it identified specific causes of low vaccination coverage and generated valuable insights directly applicable to improving the efficacy of public health initiatives. The study aimed to examine the vaccination coverage and determinants for children aged 12–23 months at community health centers in Mogadishu, Somalia. By identifying barriers to vaccination, the study aids in developing more targeted interventions and strategies to improve vaccine coverage and child health in the region.

## Methods

### Study area

This study was conducted in Mogadishu, the capital of Somalia, situated in the coastal Benadir region, a densely populated area with an estimated 2.388 million residents. The city features a mix of residential and commercial zones and is undergoing infrastructure recovery.

### Study design and population

The study employed a descriptive cross-sectional approach and was conducted at two government-owned community health centers in the Hodon and Waaberi districts of Mogadishu from March 25 to June 15, 2024. The reason for choosing these two MCHs was feasibility and accessibility. The study focused on children aged 12–23 months whose mothers and caregivers were receiving antenatal care at these community health centers. Both facilities recruited an equal proportion of study participants and experienced a combined daily client load of approximately 50–55 patients seeking vaccinations and related antenatal care services.

### Selection criteria

The study included all mothers with children aged 12–23 months who visited the Health Center during the data collection period. Mothers whose children were aged above 23 months or below 11 months at the time of data collection were excluded from the study.

### Sample size determination

The sample size was determined using the single population proportion formula and was expressed as:

$$N=Z^{2}pq/d^{2}$$

Where:

- *N*: stands for the required sample size.
- *Z*^2^: Reflects the 95% confidence level, with a value of 1.96.
- *p*: Denotes the expected proportion of the population, The proportion used for the calculation was based on a previous study, which reported a value of 0.45 (14).
- *d*: Indicates the acceptable margin of error, which is 5%.

By substituting the values into the formula, the calculations are as follows:

$$N=Z^{2}pq/d^{2}$$

$$N=\frac{{\left(1.96\right)}^{2}\times 0.45\times 0.55}{0.05^{2}}$$

N=380

To ensure adequate statistical power and address potential data loss, a final sample size of 417 participants was established, considering a 10% anticipated non-response rate. Participants were selected using Systematic sampling, enrolling every third of eligible mothers and caregivers presenting at participating community health centers until the target sample size was reached.

### Data collection

Data were collected using a structured questionnaire. The questionnaire was adapted from relevant published literature, contextualized to the local situation, and translated into Somali. Data collection was facilitated by trained staff to collect accurate and reliable data from the principal investigator. The questionnaire was pretested on 5% of the sample not included in the main study. Data collected included socio-demographic factors and health system factors. Data collected each day were checked visually for completeness by the supervisors and the principal investigator.

## Study variables

### Dependent Variable

The primary outcome variable for this study was child vaccination status. This variable was categorized as: fully vaccinated or partially vaccinated.

### Independent variables

This study examined a range of individual- and community-level factors as independent variables. These included; Maternal age (20–26, 27–33, 34–40 years), marital status (Single, Married, Divorced, Widowed), maternal level of education (No education, Primary, Secondary, Higher), maternal employment status (Employed, Unemployed), household monthly income in US dollars (100–200, 200–300, 300–400, 400–500), child's age (12–14, 15–17, 18–20, 21–23 months), child's sex (Male, Female), birth order (First or second, Third or fourth, Fifth and above), distance to health provider in kilometers (1–4, 5–7, 8–10), healthcare availability (Yes, No), cost affordability (Yes, No), vaccine acceptance (Yes, No), number of ANC visits (None, 1–3, 4+), and place of delivery (Health Facility, Home).

## Operational definitions

- **Fully vaccinated:** A child is considered fully vaccinated if they have received BCG, Hepatitis B, OPV/IPV, DTP, Hib, PCV, Rotavirus, Measles/MMR, and Yellow Fever vaccines.
- **Partially vaccinated:** A child who had received some of the nine basic vaccines or who had not received all of the full doses.
- **Antenatal Care (ANC):** is the routine care given to pregnant women to ensure maternal and fetal well-being before childbirth.
- **Community health center:** is a primary healthcare center (PHC) that serves as the first point of contact for individuals seeking medical care within the Hodon and Waaberi districts

### Data analysis

Statistical software (SPSS version 20) was used for data analysis. This included descriptive statistics to summarize participants' socio-demographic characteristics and vaccination rates. Bivariate and multivariate logistic regression analyses were employed to identify factors significantly associated with vaccination coverage. The results are presented as crude odds ratios (COR) and adjusted odds ratios (AOR), with corresponding 95% confidence intervals (CI). Statistical significance was set at a *p*-value of less than 0.05.

### Ethics clearance

This study was conducted by the Declaration of Helsinki. Ethics approval was obtained from the Jamhuriya Research Ethics Committee, with research Approval Ref. No. JURECOO50/FMHS0031/093023 and written informed consent was obtained from all participants or their legal guardians before data collection.

## Results

A total of 417 participants with diverse sociodemographic backgrounds were included in the study. All eligible mothers agreed to be included. The majority of mothers were aged 20–26 years (45.8%), followed by those aged 27–33 years (26.4%) and 34–40 years (27.8%). Most participants were married (78.4%) and a significant proportion had no formal education (60.2%). Additionally, the majority of mothers were unemployed (81.1%). Household monthly incomes were distributed as follows: 23.7% of households earned between $100–$200, 27.1% earned between $200–$300, 25.4% earned between $300–$400, and 23.7% earned between $400–$500.

Regarding children, the majority were aged 12–14 Months (50.6%), with a nearly equal distribution of males (48.0%) and females (52.0%). Birth order varied, with 30.7% being first or second born, 44.8% being third or fourth born, and the remainder fifth or above in birth order, as shown in Table 1.

**Table 1:** Sociodemographic characteristics of participants.

Variables	Frequency	Percent
Age of the mother		
20–26	191	45.8
27–33	110	26.4
34–40	116	27.8
Marital status		
Single	5	1.2
Married	327	78.4
Divorced	65	15.6
Widowed	20	4.8
Level of education		
No education	251	60.2
Primary	101	24.2
Secondary	53	12.7
Higher	12	2.9
Employment of the mother		
Employed	79	18.9
Unemployed	338	81.1
Household monthly income		
100–200	99	23.7
200–300	113	27.1
300–400	106	25.4
400–500	99	23.7
Age of child		
12–14	211	50.6
15–17	157	37.6
18–20	34	8.2
21–23	15	3.6
Sex of child		
Male	200	48.0
Female	217	52.0
Birth order		
First or second	128	30.7
Third or fourth	187	44.8
Fifth and above	102	24.5

### Level of vaccination coverage

Vaccination levels among the children in this study revealed that 53% were fully vaccinated, and 47% were partially vaccinated, as presented in Table 2. Fully vaccinated refers to children who received all the vaccines recommended for their age, while partially vaccinated refers to children who received some, but not all, of the recommended vaccines. Notably, none of the children in the sample were completely unvaccinated. All children in the sample received the BCG and OPV-0 vaccines, indicating universal uptake of these vaccines. High levels of coverage were observed for Pentavalent-1 (98.6%) and Measles-1 (98.3%) vaccines. However, lower proportions of children received subsequent doses of the vaccines, with coverage decreasing from OPV-1 (82.5%), and Pentavalent-2 (81.6%), and to Measles-2 (83.3%), OPV-2 (68.9%), Pentavalent-3 (67.9%), OPV-3 (52.6%), and IPV (53.8%). These results indicate a high initial uptake of the primary vaccinations, but lower coverage of subsequent or additional vaccines.

**Table 2:** Association between sociodemographic and partial vaccination status of the children.

Variables	Vaccination status		COR (95% CI)	AOR (95% CI)	*P*-value
	Fully vaccinated (n%)	Partial vaccinated (n%)			
Age of the mother					
20–26	89 (46.6%)	102 (53.4%)	1.875 (1.171–3.002)	1.763 (1.011–3.072)	0.135
27–33	60 (54.5%)	50 (45.5%)	1.364 (0.802–2.318)	1.432 (0.806–2.544)	
34–40	72 (62%)	44 (38%)	1	1	
Marital status					
Single	3 (60%)	2 (40%)	0.667 (0.091–4.889)	0.699 (0.084–5.823)	0.721
Married	176 (53.8%)	151 (46.2%	0.858 (0.348–2.117)	0.608 (0.230–1.608)	
Divorced	32 (49.2%)	33 (50.8%)	1.031 (0.378–2.810)	0.743 (0.254–2.174)	
Widowed	10 (50%	10 (50%)	1	1	
Level of education					
No education	143 (57%)	108 (43%)	0.539 (0.167–1.746)	0.374 (0.105–1.330)	0.085
Primary	52 (51.5%)	49 (49.5%)	0.673 (0.200–2.262)	0.447 (0.122–1.634)	
Secondary	21 (39.6)	32 (60.4%)	1.088 (0.305–3.887)	0.825 (0.218–3.114)	
Higher	5 (41.6%)	7 (58.3%)	1	1	
Occupation					
Housewife	46 (58.2%)	33 (41.8%)	0.770 (0.469–1.264)	0.630 (0.354–1.121)	0.116
Businesswoman	175 (51.8%)	163 (48.2%)	1	1	
Household income					
100–200	51 (51.5%)	48 (48.5%)	2.065 (1.157–3.685)	2.058 (1.123–3.771)	0.002
200–300	59 (52.2%)	54 (47.8%)	2.008 (1.144–3.524)	2.223 (1.229–4.021)	
300–400	43 (40.5%)	63 (59.5%)	3.214 (1.808–5.712)	3.247 (1.784–5.910)	
400–500	68 (68.7%)	31 (31.3%)	1	1	
Age of child					
12–14	115 (54.5%)	96 (45.5%)	1.252 (0.430–3.643)	1.096 (0.353–3.401)	0.948
15–17	81 (40%)	76 (48.4%)	1.407 (0.478–4.142)	1.209 (0.385–3.799)	
18–20	16 (47%)	18 (53%	1.687 (0.492–5.791)	1.297 (0.351–4.794)	
21–23	9 (60%)	6 (40%)	1	1	
Sex of child					
Male	100 (50%)	100 (50%	1.260 (0.857–1.853)	1.245 (0.827–1.872)	0.294
Female	121 (55.8%)	96 (44.2%)	1	1	
Birth order					
First or second	64 (50%)	64 (50%	1.615 (0.952–2.741)	0.968 (0.504–1.857)	0.971
Third or fourth	94 (50.2%)	93 (49.8%)	1.598 (0.978–2.613)	1.030 (0.590–1.797)	
Fifth and above	63 (61.8%)	39 (38.2%)	1	1	
Overall vaccination status	221 (53%)	196 (47%)			

### Factors associated with vaccination coverage

The analysis found that higher household income is associated with an increased odds of partial vaccination status. Families with an income of $300–$400 $300–$400 monthly are most likely to be partially vaccinated (AOR: 3.247, 95% CI: 1.784–5.910, *p* = 0.002), compared to those with lower incomes. Other sociodemographic factors, including age, marital status, level of education, occupation, and the age and sex of the child, were not significantly associated with vaccination status according to this study, as shown in Table 2.

The analysis revealed that children of mothers who had no antenatal care (ANC) visits were significantly less likely to be fully immunized (AOR = 20.075, 95% CI: 7.385–54.572, *p* = 0.001) than those whose mothers had four or more ANC visits. Other factors, including distance to healthcare providers, availability of healthcare providers, cost affordability, vaccine acceptance, and place of delivery, were not statistically significant, as shown in Table 3.

**Table 3:** Factors associated with the vaccination status of children.

Variables	Vaccination level		COR ratio with 95% CI	AOR ratio with 95% CI	*P* value
	Fully vaccinated (n%)	Partial Vaccinated (n%)			
Distance of health provider (km)					
1–4	94 (52%)	87 (48%)	1.628 (0.947–2.796)	0.892 (0.458–1.738)	0.135
5–7	76 (48.7)	80 (51.3%)	1.851 (1.064–3.220)	1.523 (0.746–3.109)	
8–10	51 (63.8%)	29 (36.2%)	1	1	
Healthcare provider availability					
Yes	168 (53.2%)	148 (46.8%)	0.973 (.621–1.524)	1.133 (0.665–1.930)	0.647
No	53	48	1	1	
Cost affordability					
Yes	201 (52%)	185 (48%)	1.673 (0.781–3.587)	1.805 (0.710–4.587)	0.214
No	20 (64.5%)	11 (35.5%)	1	1	
Vaccine acceptance					
Yes	216 (52.8%)	193 (47.2%)	1.489 (0.351- 6.314)	1.490 (0.284–7.813)	0.637
No	5 (62.5)	3 (37.5%)	1	1	
Number of ANC visits					
None	50 (31.8%)	107 (68.2%)	18.404 (6.872–49.286)	20.075 (7.385 -54.572)	0.001
1–3	34 (33.7%)	67 (62.3%)	16.947 (6.149–46.711)	19.631 (6.972–55.274)	
4–5	94 (84.7%)	17 (15.3%)	1.555 (0.539–4.491)	1.534 (0.527–4.462)	
above 5	43 (89.6%)	5 (10.5%)	1	1	
Place of delivery of child					
Health facility	166 (53%)	147 (47%)	0.994 (0.637–1.550)	1.068 (.613–1.863	0.816
Home	55 (52.9%)	49(47.1%)	1	1	

## Discussion

This study examined the vaccination status and determinants for children aged 12–23 months at community health centers in Mogadishu, Somalia. Our study found that 53% of children were fully immunized, while 47% were partially vaccinated. This rate is higher than that observed in previous studies from Galkayo and Mogadishu, where vaccination rates were lower, but still lower than those reported in Hargeisa (1, 13, 14). Additionally, the findings revealed a significant association between household income and partial vaccination status, showing that families with an income between $300 and $400 had a notable most likely of partial vaccination status (AOR: 3.247, 95% CI: 1.784–5.910, *p* = 0.002). This result is different from previous research which consistently demonstrates that higher household income facilitates access to healthcare services, including vaccinations (14). This discrepancy may be due to methodological differences; our study is a cross-sectional analysis conducted at health centers, while previous research was largely household-based. Furthermore, community health centers often provide supplemental nutrition and childcare resources alongside vaccinations, which may incentivize vaccination adherence among families with low incomes.

In contrast, our study did not find significant associations with sociodemographic factors like maternal literacy and birth order, unlike other research that links higher maternal literacy with better vaccination coverage, indicating that educated parents are generally more informed and adept at accessing healthcare (1, 13, 14, 16–19, 22). This may be due to specific characteristics of our study population or differences in how these factors interact with vaccination coverage in our context.

Our analysis further revealed that children whose mothers had no antenatal care (ANC) visits were significantly less likely to be fully immunized compared to those whose mothers had four ANC visits (AOR = 5.202, *p* = 0.001). This is consistent with previous studies that found antenatal care attendance significantly influences vaccination rates, with children born in hospitals being more likely to receive full vaccination (13, 20). However, this study did not find some factors to be significantly associated with vaccination coverage as reported in other studies. Discrepancies may arise from differences in study populations, timing of data collection, definitions of vaccination status, study methodologies, or unmeasured factors.

This finding highlights the critical role that ANC visits play in ensuring complete vaccination, as they provide an opportunity for healthcare providers to educate mothers on the importance of vaccines and to ensure the timely administration of vaccines during healthcare visits.

### Limitations of the study

Limitations of this study include the cross-sectional design limits the ability to establish causality between identified factors and vaccination status. Finally, convenience sampling was used to recruit participants, which may have introduced selection bias and thus limited the representativeness of the results.

## Conclusion

This study found a low full vaccination rate of 53%. Higher vaccination rates correlated with higher income and consistent antenatal care (ANC). We recommend increasing ANC access, addressing socioeconomic barriers, and strengthening community outreach. Future research is essential to understand and address the unique barriers to vaccination experienced by low-income communities, while also prioritizing the development and implementation of innovative community-led engagement approaches.

## Data Availability

The raw data supporting the conclusions of this article will be made available by the authors, without undue reservation.
